# Genetic modification of alternative respiration in *Nicotiana benthamiana *affects basal and salicylic acid-induced resistance to potato virus X

**DOI:** 10.1186/1471-2229-11-41

**Published:** 2011-02-28

**Authors:** Wing-Sham Lee, Shih-Feng Fu, Jeanmarie Verchot-Lubicz, John P Carr

**Affiliations:** 1 Department of Plant Sciences, University of Cambridge, Downing Street, Cambridge CB2 3EA, UK; 2 Oklahoma State University, Department of Entomology and Plant Pathology, 127 Noble Research Center, Stillwater, OK 74078, USA

## Abstract

**Background:**

Salicylic acid (SA) regulates multiple anti-viral mechanisms, including mechanism(s) that may be negatively regulated by the mitochondrial enzyme, alternative oxidase (AOX), the sole component of the alternative respiratory pathway. However, studies of this mechanism can be confounded by SA-mediated induction of RNA-dependent RNA polymerase 1, a component of the antiviral RNA silencing pathway. We made transgenic *Nicotiana benthamiana *plants in which alternative respiratory pathway capacity was either increased by constitutive expression of AOX, or decreased by expression of a dominant-negative mutant protein (AOX-E). *N. benthamiana *was used because it is a natural mutant that does not express a functional RNA-dependent RNA polymerase 1.

**Results:**

Antimycin A (an alternative respiratory pathway inducer and also an inducer of resistance to viruses) and SA triggered resistance to tobacco mosaic virus (TMV). Resistance to TMV induced by antimycin A, but not by SA, was inhibited in *Aox *transgenic plants while SA-induced resistance to this virus appeared to be stronger in *Aox-E *transgenic plants. These effects, which were limited to directly inoculated leaves, were not affected by the presence or absence of a transgene constitutively expressing a functional RNA-dependent RNA polymerase (MtRDR1). Unexpectedly, *Aox*-transgenic plants infected with potato virus X (PVX) showed markedly increased susceptibility to systemic disease induction and virus accumulation in inoculated and systemically infected leaves. SA-induced resistance to PVX was compromised in *Aox*-transgenic plants but plants expressing AOX-E exhibited enhanced SA-induced resistance to this virus.

**Conclusions:**

We conclude that AOX-regulated mechanisms not only play a role in SA-induced resistance but also make an important contribution to basal resistance against certain viruses such as PVX.

## Background

Salicylic acid (SA) is an important defensive signal in plants that is required for elicitor-triggered immunity and the establishment of systemic acquired resistance (SAR)[1-8]. Plants exhibiting SAR possess an enhanced state of protection against a broad spectrum of pathogens, including viruses, oomycetes, fungi and bacteria [3,5]. SA inhibits various phases of the viral life cycle including replication, cell-to-cell movement and systemic movement. However, the precise effects of SA can differ between various host-virus combinations [9-16].

RNA silencing (also known as post-transcriptional gene silencing) is thought likely to contribute to SA-induced virus resistance although it is unlikely to be the only mechanism involved [17]. RNA silencing is a sequence-specific mechanism regulating the synthesis, stability, and translatability of mRNA molecules that is guided by small RNA molecules in the size range 21-26 nt [reviewed in ref. [18]]. The involvement of RNA silencing in SA-induced virus resistance was first suggested by the discovery that SA can induce expression of a component of the RNA silencing machinery, RNA-directed RNA polymerase 1 (RDR1) [19]. RDR1 may also contribute to defense through regulation of host mRNAs encoding other defensive factors, for example those involved in jasmonic acid-induced defenses [19-24]. Additional evidence for a role for RNA silencing in SA-mediated defense arose from studies showing that viral silencing suppressor proteins, for example the cucumber mosaic virus (CMV) 2b protein or HC-Pro encoded by potyviruses, interfere with SA-mediated signaling and SA biosynthesis [25-28].

There is evidence that mitochondrial signaling processes regulate some aspects of SA-induced virus resistance [discussed by [1,3]]. Reactive oxygen species (ROS) are constantly generated within mitochondria as by-products of respiratory electron transport chain activity [29-31]. Perturbation in this ROS pool or in mitochondrial redox can function in intracellular signal transduction and, through the poorly understood process of mitochondrial retrograde regulation, affect the pattern of nuclear gene expression [31-35]. This form of signaling is influenced by the alternative oxidase (AOX). AOX is a mitochondrial enzyme that is the sole component of the alternative respiratory pathway. The functions of the alternative respiratory pathway include negative regulation of mitochondrial ROS production and maintenance of primary metabolism under stress conditions [30,36-40].

Evidence supporting an additional role for mitochondrial signaling and AOX in virus resistance includes observations that non-toxic levels of respiratory inhibitors such as antimycin A or cyanide induce resistance against several plant viruses [9,12,13,15,16,41,42], and that SA, which is a weak cytochrome pathway inhibitor, induces *Aox1a *gene expression [36,43,44]. Murphy and associates [45] found that expression of an AOX coding sequence by a TMV-derived expression vector enhanced its spread in *N. benthamiana *plants. Gilliland and colleagues [20] found that in directly inoculated leaves, SA- and antimycin A-induced resistance to TMV was transiently enhanced in transgenic plants that had decreased alternative respiratory pathway capacities. However, in transformed tobacco plants that had increased alternative respiratory pathway capacities due to constitutive expression of an *Aox1a *transgene, the induction of resistance to TMV by antimycin A was inhibited, while SA-induced resistance was not [20].

To explain these results, it was proposed that SA and antimycin A both stimulate a signaling pathway that is negatively-regulated by AOX. However, RDR1, a factor in resistance to TMV [19], is also inducible by SA but not by antimycin A [20,46]. Since *RDR1 *gene expression is not affected by AOX, it was suggested that this is why an increase in alternative respiratory pathway capacity inhibits antimycin A-induced resistance but does not completely inhibit SA-induced resistance to TMV [20,46]. To further investigate the AOX-regulated mechanism of resistance to viruses we have used the experimental host plant *N. benthamiana*. This is highly susceptible to a wide range of viruses, which is explained to some extent by the fact that its *RDR1 *ortholog, *NbRDR1m*, encodes an inactive enzyme [22]. Thus, *N. benthamiana *provides a natural mutant background for exploring the effect of AOX on plant responses to virus infection without the potentially confounding presence of active RDR1.

## Results

### Construction and characterization of stably transformed *N. benthamiana *plants with modified alternative respiratory pathway capacity

To increase or decrease alternative respiratory pathway capacity, *N. benthamiana *plants (non-transgenic or already expressing an *RDR1 *transgene derived from *Medicago truncatula*: 22) were transformed with, respectively, either a wild-type tobacco *Aox1a *cDNA, or a mutant sequence, *Aox-E *[45]. *Aox-E *encodes an inactive version of AOX (AOX-E) to act as a dominant negative mutant [45,47]. Transgenes were fused to an enhanced cauliflower mosaic virus 35S promoter to drive constitutive high-level expression (Additional File 1). Transgene expression and the respiratory characteristics of transgenic plant lines were examined [20,40,45] and lines with up- or down-regulated alternative respiratory capacity were selected for subsequent experiments (Table 1).

**Table 1:** Alternative respiratory pathway capacity in transgenic plant lines expressing AOX or AOX-E that were used in this study

		Oxygen Consumption Rates (nmol O_2 _min^-1^/10^6 ^cell)^2^					
**Plant Line^1^**	**Transgene(s)**	**Control**	**Antimycin A**	**Antimycin A + SHAM**	**Corrected Control^3^**	**AP Capacity^4^**	**n**

NT	-	1.86 ± 0.08	1.43 ± 0.01	0.04 ± 0.04	1.82	1.39	8

AOX1	35S:*Aox1a*	5.35 ± 0.36	6.08 ± 0.68	0.79 ± 0.13	4.57	5.30	3

AOX3	35S:*Aox1a*	3.38 ± 0.33	4.62 ± 0.54	0.54 ± 0.11	2.83	4.07	3

AOX-E3	35S:*Aox-E*	2.03 ± 0.28	0.77 ± 0.13	0.39 ± 0.06	1.64	0.39	4

AOX-E5	35S:*Aox-E*	1.68 ± 0.11	0.73 ± 0.11	0.18 ± 0.02	1.50	0.55	3

Empty vector control	-	2.18 ± 0.27	1.34 ± 0.11	0.29 ± 0.03	1.89	1.05	4

RD/AOX2	35S:*Aox1a/ *35S:*MtRDR1*	1.99 ± 0.06	2.47 ± 0.13	0.065 ± 0.003	1.93	2.41	3

RD/AOX22	35S:*Aox1a/ *35S:*MtRDR1*	2.08 ± 0.03	2.55 ± 0.01	0.105 ± 0.024	1.98	2.45	3

Notes.

^1^Leaf cells isolated from non-transgenic (NT) *Nicotiana benthamiana *plants, transgenic plants expressing *Aox1a *or *Aox-E *sequences under the control of the cauliflower mosaic virus 35S promoter, plants harboring a 35S:MtRDR1 transgene super-transformed with the 35S:*Aox1a *transgene, or from plants transformed with an 'empty' transformation vector were placed in an oxygen electrode. The number (n) of independent oxygen electrode experiments (each of which comprised three technical replicates) carried out for each plant line is indicated.

^2^Oxygen consumption rates (± standard error of the mean) were measured in the absence (Control) or presence of 25 μM antimycin A (an inhibitor of the cytochrome pathway) or 25 μM antimycin A plus 2 mM salicylhydroxamic acid (SHAM: an inhibitor of the alternative respiratory pathway). Rates determined when both inhibitors were present, gave a measure of residual, i.e. non-respiratory, oxygen consumption.

^3^Last two columns: residual values were used to derive corrected values for the control oxygen consumption rates, and oxygen consumption rates in the presence of antimycin A, which gives the alternative respiratory pathway (AP) capacity for each line.

^4^AP capacity in plant cells expressing the Aox or Aox-E transgenes were significantly different from that observed in cells from non-transgenic plants (p<0.05: Student's t-test).

### Altering alternative respiratory pathway capacity affected basal resistance to potato virus X

**Figure 1:**
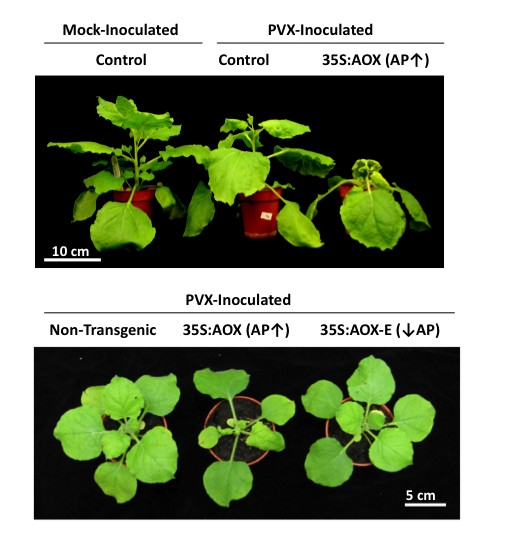
**Increasing alternative respiratory pathway capacity exacerbates disease symptoms in potato virus X infected plants**. Five-week-old non-transgenic plants (NT), or plants transformed with an 'empty' transformation vector (Control), and plants of transgenic lines with increased alternative respiratory pathway capacity (AP↑) or decreased AP capacity (AP↓) inoculated on one of the lower leaves with purified PVX (0.5 μg/ml) and photographed 11 days later. Compared to the effects of PVX infection on non-transgenic, transgenic control or *AOX-E*-transgenic plants with decreased AP capacity, increased stunting and deformation of upper leaves was evident in PVX-inoculated plants belonging to *Aox-*transgenic plants with increased AP capacity.

**Figure 2:**
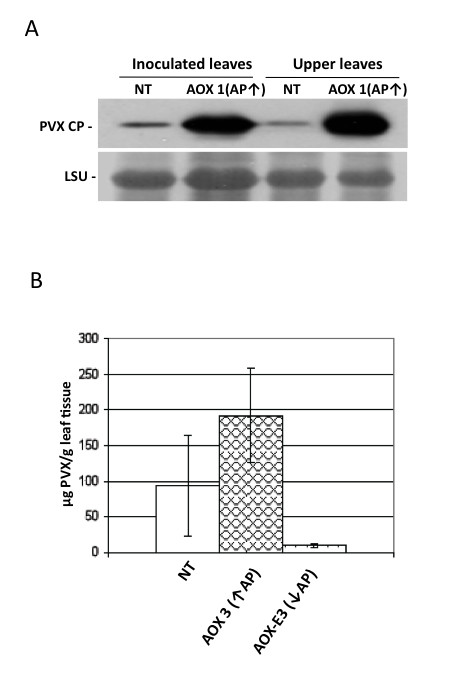
**Susceptibility of plants to potato virus X accumulation in directly inoculated and systemically infected leaves is altered by modification of alternative respiratory pathway capacity**. (A) Semi-quantitative analysis of virus accumulation in PVX-inoculated non-transgenic and *Aox-*transgenic plants by immunoblot analysis using anti-PVX coat protein (CP). This showed that virus accumulation was higher in directly-inoculated and systemically-infected leaves of plants with increased AP capacity than in corresponding tissues of non-transgenic plants. (B) Quantitative analysis (enzyme-linked immunosorbent assay) of PVX coat protein accumulation in non-transgenic (NT) plants and plants belonging to transgenic lines with increased (↑) or decreased (↓) AP capacity. Systemically-infected (i.e., upper, not directly-inoculated) leaf tissue was harvested at 14 days post-inoculation. Tissue from the fourth leaf above the inoculated leaf was taken in each case. Using a standard curve normalized with purified PVX, mean virus concentrations per gram of leaf tissue were determined and the data from one experiment, out of a total of three independent experiments, are plotted above showing a significant (t-test, *p *< 0.05) difference in PVX accumulation in plants with decreased AP capacity compared with those with increased AP capacity. The number of samples was six for each plant line (one sample = one plant) in each treatment group. Error bars represent mean ± standard error of the mean.

**Figure 3:**
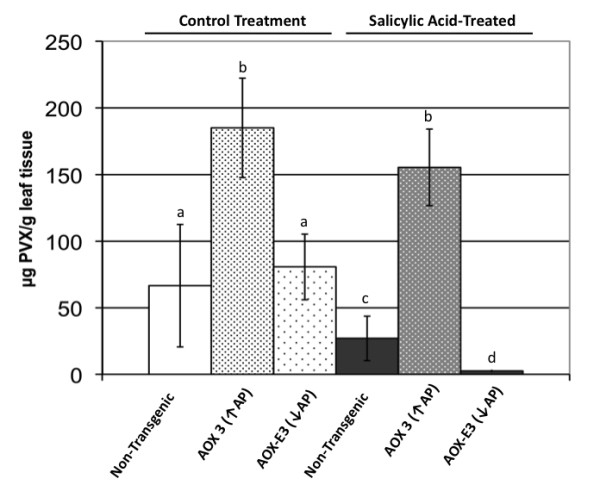
**The effects of altering alternative respiratory pathway capacity on salicylic acid (SA)-induced resistance to potato virus X (PVX)**. Plants were pre-treated by spraying with a solution of SA or a control solution for four days prior to inoculation with PVX. Samples from the fourth leaf above the directly PVX-inoculated leaves of plants were harvested at 28 days post-inoculation and processed for an enzyme-linked immunosorbent assay using anti-PVX coat protein serum. Mean accumulation (μg PVX per gram of leaf tissue) was determined using a standard curve and the data from one experiment, out of a total of three independent experiments, are plotted above. The number of samples was six for each plant line (one sample = one plant) in each treatment group. Error bars represent mean ± standard error of the mean. Accumulation of virus was significantly different between non-transgenic and AOX 3 *Aox-*transgenic plants (p = 0.044) and between SA-treated and SA-treated AOX3 *Aox*-transgenic plants (p = 0.020). Mean titer values labelled with the same lower case letter were not significantly different from each other (t-test, *p *> 0.05).

Modifying alternative respiratory capacity had dramatic effects on disease symptoms in PVX-infected plants (Figure 1) and on virus accumulation in directly-inoculated and systemically infected leaves (Figure 2A). PVX caused mild symptoms in non-transgenic *N. benthamiana *plants but caused stunting and leaf curling symptoms in *Aox*-transgenic plants with increased alternative pathway capacities (Figure 1). Accumulation of PVX coat protein was higher in *Aox*-transgenic plants with than in non-transgenic plants (Figure 2A). Decreasing the alternative pathway capacity by expression of AOX-E did not appear to affect symptom development following infection with PVX (Figure 1). At 14 days post-inoculation significantly lower levels of virus had accumulated in plants expressing AOX-E than those expressing AOX (*p *= 0.02). Although at 14 days post-inoculation less virus accumulated in plants expressing AOX-E than in non-transgenic plants these differences were not statistically significant (t-test, *p *= 0.145) (Figure 2B) and were not observed consistently at later time points (Figure 3). These data show that modulating alternative respiratory capacity affects PVX accumulation.

### Salicylic acid-induced resistance to PVX is modified in plants with increased and decreased alternative respiratory pathway capacity

Following treatment with SA, PVX coat protein accumulation was monitored in transgenic plants from lines with increased or decreased alternative respiratory capacities at 28 days post-inoculation. Treatment with SA induced resistance to PVX in non-transgenic *N. benthamiana *but the treatment did not induce resistance to the virus in *Aox*-transgenic plants (Figure 3). However, as an inducer of resistance to PVX, SA was markedly more effective in these plants than in non-transgenic or *Aox*-transgenic plants, with virus accumulation decreased to barely detectable levels (Figure 3). These data show that in *N. benthamiana *plants, suppression of alternative respiratory pathway capacity combined with SA treatment led to greater PVX resistance and that SA-induced resistance to PVX was compromised by enhancement of alternative respiratory pathway capacity. Thus, for PVX, AOX-modulated defensive signalling is the predominant factor in the regulation of SA-induced resistance.

### Altering alternative respiratory pathway capacity affects chemical induction of resistance to tobacco mosaic virus in *N. benthamiana*

Pre-treatment of non-transgenic *N. benthamiana *plants with SA inhibited TMV-induced symptom development in systemically infected tissues. However, modifying alternative respiratory pathway capacity in transgenic plants did not affect the timing or appearance of systemic disease symptoms induced by TMV regardless of whether or not the transgenic plants had been treated with SA (data not shown), similar to results in tobacco [20].

**Figure 4:**
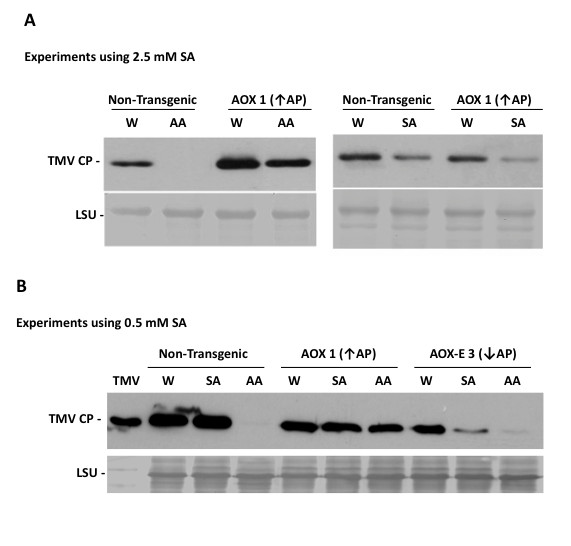
**Modifying alternative respiratory pathway capacity affects chemical induction of resistance to tobacco mosaic virus (TMV) in inoculated leaves**. TMV coat protein (CP) accumulation examined 72 hours post-inoculation by immunoblotting using anti-TMV CP serum. Leaves of non-transgenic *N. benthamiana *plants, plants transformed with an 'empty' transformation vector (Control), or T_2 _generation transformed plants expressing wild-type *AOX *(AOX1) or mutant *AOX-E *(AOX-E3) transgenes were infiltrated with solutions of salicylic acid (SA), antimycin A (AA), or water (W) containing 0.05% (v/v) ethanol (equivalent to the ethanol used to dissolve SA and AA before dilution). Within 10 min of infiltration, leaves were inoculated with TMV (0.05 μg/ml in water). Arrows denote transgenic lines with increased (↑) or decreased (↓) alternative respiratory pathway (AP) capacities. A. Induction of resistance to TMV by AA (2 μM) in directly inoculated leaf tissue was inhibited in plants with increased AP capacity. However, induction of resistance to TMV by SA (2.5 mM) in directly inoculated leaf tissue was not appreciably inhibited in plants with an increased AP capacity. B. A concentration of SA (0.5 mM) that was insufficient to induce resistance in non-transgenic leaves did induce resistance in plants with decreased AP capacity (line AOX-E3). AA at 2 μM was sufficient to induce resistance in non-transgenic leaves and in plants with decreased AP capacity, but insufficient to do so in plants with increased AP capacity. Equal loading of lanes indicated by accumulation of ribulose-1, 5-bisphosphate carboxylase/oxygenase large subunit (LSU) was revealed by Ponceau S staining of immunoblot membranes.

In contrast, modifying the alternative respiratory capacity of *N. benthamiana *plants did affect the induction of resistance by antimycin A and SA to TMV accumulation in directly inoculated leaf tissue (Figure 4). Leaves were infiltrated with a control solution or solutions of antimycin A or SA before inoculation with TMV. Viral coat protein accumulation in these leaves was detected by immunoblot analysis (Figure 4). In *Aox-*transgenic lines with increased alternative respiratory pathway capacities, antimycin A-induced resistance to TMV was inhibited (Figure 4). However, SA-induced resistance to TMV was not inhibited in these plants (Figure 4A). The concentration of SA (2.5 mM) required to induce resistance to TMV in non-transgenic *N. benthamiana *was markedly higher than the 0.5 mM concentration that is sufficient to induce resistance to TMV in tobacco [20] (Figure 4, compare panels A and B). This may reflect the greater susceptibility to virus infection that is characteristic of *N. benthamiana *[48], or that inactivation of SA by conversion to its glucoside occurs more effectively in this plant [49]. However, in transgenic plants expressing AOX-E, that have a decreased capacity for alternative respiration, 0.5 mM SA was adequate to induce resistance to TMV infection even though it did not induce resistance to TMV in non-transgenic *N. benthamiana *leaves (Figure 4B). This was also seen in three other transgenic lines expressing the AOX-E mutant protein (data not shown). Thus, as was found to be the case in tobacco [20], in *N. benthamiana *AOX activity can negatively regulate the induction by SA and antimycin A of resistance against TMV. However, the effects of altering *Aox *gene expression on TMV infection overall were markedly less striking than the effects these alterations had on PVX infection.

### The effects of altering *Aox *and *RDR1 *gene expression on chemical induction of resistance to tobacco mosaic virus in *N. benthamiana*

**Figure 5:**
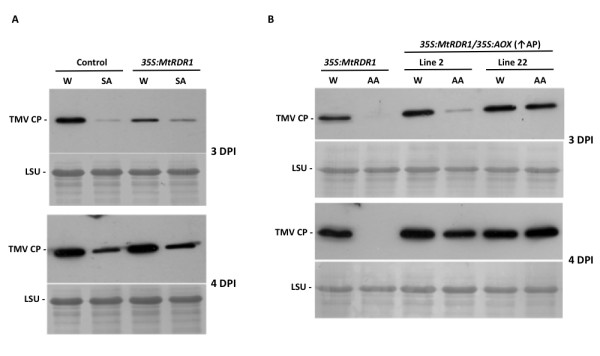
**Chemically-induced resistance to TMV in plants expressing *MtRDR1 *and *Aox-*derived transgenes**. TMV coat protein (CP) accumulation in directly-inoculated leaves was examined at 3 and 4 days post-inoculation (DPI) by immunoblot analysis of soluble proteins. (A) Leaves of *N. benthamiana *plants transformed with an 'empty' transformation vector (Control), or a *MtRDR1 *transgene fused to the cauliflower mosaic virus 35S promoter (22) were infiltrated with water [W: amended with 0.05% (v/v) ethanol] or 2.5 mM SA prior to inoculation with 0.05 μg/ml TMV U1. (B) Leaves of plants harboring the 35S:*MtRDR1 *transgene or doubly-transformed with the 35S:*MtRDR1 *transgene and an *AOX *transgene fused to the 35S promoter, from lines confirmed to have increased (↑) alternative respiratory pathway (AP) capacity, were infiltrated with water or 2 μM antimycin A prior to inoculation. Equal loading of gel lanes with protein is shown by accumulation of ribulose-1, 5-bisphosphate carboxylase/oxygenase large subunit (LSU) revealed by Ponceau S staining of the immunoblot membrane.

Previously, we hypothesized that the reason that antimycin A-induced TMV resistance, but not SA-induced resistance, is inhibited in *Aox*-transgenic transgenic plants is because SA triggers increased expression of the antiviral enzyme RDR1, while antimycin A does not induce *RDR1 *[20,46]. We investigated the effect of constitutive RDR1 expression on chemically-induced resistance to TMV using plants doubly transformed with *Aox*-derived transgenes and a transgene expressing the *M. truncatula RDR1 *sequence (*MtRDR1*). *MtRDR1*-transgenic plants are more resistant to TMV-induced disease and so, in contrast to transgenic control and non-transgenic plants, were not killed by systemic infection with TMV (Additional File 2 and ref. 22). However, the expression of *MtRDR1 *did not result in any marked effect on TMV accumulation in the directly inoculated leaves (Figure 5), suggesting that the major effect of RDR1 is to protect plants from systemic infection. Constitutive expression of *MtRDR1 *did not prolong the inhibitory effect of SA on TMV accumulation (Figure 5A), nor did it affect the characteristics of antimycin A-induced resistance to TMV. Although the suppression of antimycin A-induced resistance to TMV was slightly stronger in line 22 than in line 2, in neither of these AOX-expressing lines did the constitutive expression of MtRDR1 rescue chemically-induced resistance to the virus (Figure 5B).

## Discussion

In line with a model proposed by Singh and colleagues [46], modifying alternative respiration by constitutive expression of AOX or the dominant-negative mutant AOX-E affected the outcome of infection by viruses. For PVX, our data indicates that SA-induced resistance is regulated strongly by AOX and, unexpectedly, that this enzyme also plays a role in maintaining basal resistance to this virus. The interactions of these transgenic *N. benthamiana *plants with TMV are similar to those observed previously in tobacco plants transformed with *Aox*-derived transgenes [20,50]. Thus, as in tobacco, SA-induced resistance to TMV was enhanced in *N. benthamiana *plants with diminished alternative respiratory pathway capacity but apparently unaffected in plants with an enhanced respiratory capacity. Antimycin A-induced resistance to TMV was, respectively, enhanced or inhibited in transgenic plants in which alternative respiratory pathway capacity had been diminished or increased. However, basal resistance to TMV in *N. benthamiana *was not affected by modification of alternative respiratory capacity.

Previous studies showed that in tobacco, NtRDR1 activity contributes to the maintenance of basal resistance to PVX whilst in *N. benthamiana *it is NbRDR6, not NbRDR1, which is a major determinant of basal resistance against this virus [19,22,51]. Our data suggests that in *N. benthamiana *the defensive signal transduction pathway regulated by AOX also plays an important role in basal resistance to PVX. This result was unexpected since evidence from Gilliland and colleagues [20] had indicated that AOX-regulated defensive signaling plays no discernable role in basal resistance to TMV.

The result was further surprising since PVX, like TMV, is a positive-strand RNA virus [52]. Thus, these viruses replicate in a similar fashion, although they have differing strategies for gene expression and cell-to-cell movement [reviewed by [53]]. It may be that TMV is more effective than PVX at subverting or inhibiting the antiviral mechanisms that are regulated by AOX. TMV, for example, appears to be able to interfere with a wide range of SA-mediated responses through interaction between its replicase proteins and a transcription factor that regulates basal defense [54]. Alternatively, it may be that PVX is able to evade some of the SA-induced resistance mechanisms that inhibit TMV infection. For instance, although the accumulation of TMV and PVX was inhibited by NtRDR1, the RDR1 of *Medicago truncatula *inhibited TMV accumulation but PVX appears to be unaffected by this RDR1 ortholog [19,22].

Another possibility is that PVX, but not TMV, elicits a reaction similar or analogous to PAMP-triggered immunity. PAMPs or pathogen-associated molecular patterns are chemical signatures produced by cellular pathogens (bacteria, fungi, etc) that are perceived by receptor-like kinases, resulting in the triggering of localized defense responses including the generation of ROS [55]. These localized defense responses can underlie non-host resistance (in which plants are not susceptible to the pathogen) or provide some minimal level of resistance to infection (basal resistance) in susceptible plants. Typically, PAMPs are quite generic in their nature and for bacterial pathogens include fragments of flagellin or translation factors [56]. Baurès and colleagues [57] have theorized that in PVX and other potexviruses a highly conserved amino acid sequence or folded structure in the coat protein might function as a PAMP. It is also known that the PVX coat protein interacts with a factor, the RanGAP2 protein, which may act as a target or decoy for the PVX coat protein and (in plants harbouring the *Rx *resistance gene) may participate in triggering resistance [58-60]. It is possible that interactions of the PVX coat protein with an unknown receptor, or with RanGAP2 in susceptible plants (such as the *N. benthamiana *used in this study), result in induction of basal resistance mechanisms via the production of ROS. Maxwell and colleagues [36] showed that AOX can affect ROS levels throughout the cell, so it could be that this signaling mechanism is affected in plants with modified alternative respiration. Possibly consistent with this idea are observations showing that potexvirus infections are associated with complex changes in ROS and in the activity of anti-oxidant enzymes [61].

**Figure 6:**
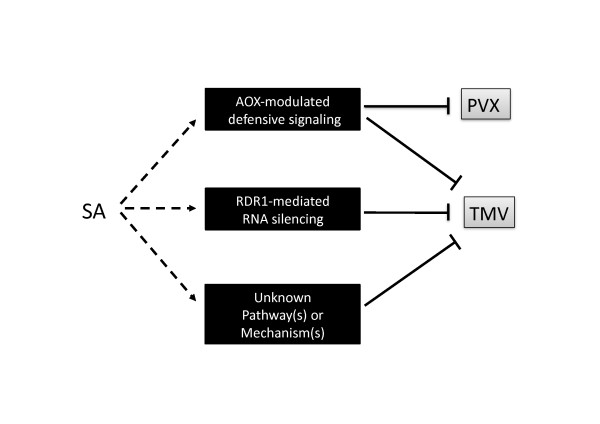
**Induction of virus resistance by salicylic acid**. Based on our data we propose that salicylic acid (SA)-mediated signaling (dashed arrows) induces several distinct mechanisms that inhibit (solid blunt-ended lines) virus infection. For some viruses, exemplified by potato virus X (PVX), anti-viral mechanisms regulated by alternative oxidase (AOX) play a predominant role. Other viruses, exemplified by tobacco mosaic virus (TMV), are affected not only by AOX-modulated mechanisms and RDR1-mediated antiviral RNA silencing but also by other, unknown mechanisms.

Our rationale for using *N. benthamiana *in this study was that the *NbRDR1m *gene encodes a protein that lacks RDR activity [22]. Thus, it was envisaged that we would be able to observe the effects of modifying alternative respiratory capacity on the effectiveness of SA-induced resistance to TMV without interference from RDR1 activity. However, we found that although antimycin A-induced resistance to TMV was affected in plants with either increased, or decreased, alternative respiratory capacity, SA-induced resistance to this virus was only affected (enhanced) in plants with a decreased capacity. The results are similar to those seen in transgenic tobacco plants with increased alternative pathway capacities [20]. Performing experiments with plants expressing MtRDR1 constitutively showed that although these plants are more resistant to systemic TMV-induced disease, the expression of this enzyme has no detectable effects on TMV accumulation in the directly-inoculated leaves or on chemically induced resistance in these tissues. Thus, our hypothesis that SA-induced resistance to TMV in tobacco could be a result of the combined action of AOX-regulated mechanisms and RDR1-mediated viral RNA turnover was overly simplistic. Our results with *N. benthamiana *do not rule out a role for RDR1 activity in SA-induced resistance to this virus, or a role for AOX as a regulator of this resistance. But the results indicate that additional, unknown factors also participate in SA-induced TMV resistance (Figure 6).

## Conclusions

The study revealed that for PVX, SA-induced resistance is much simpler in its execution than SA-induced resistance to TMV. For PVX, AOX-modulated defensive signaling is the predominant factor in controlling SA-induced resistance. In conclusion, the results of this study show that AOX-regulated signaling constitutes an important part of plant antiviral resistance. However, it is also clear that the phenomena of SA-induced resistance and basal resistance to viruses both result from the operation of multiple antiviral mechanisms that do not inhibit all viruses to an equal extent.

## Methods

### Plant Growth Conditions

Seeds of *Nicotiana benthamiana *(Domin.) non-transgenic and transgenic plant lines were, as appropriate, germinated on soil or under sterile conditions on 1% (w/v) agar containing Murashige and Skoog medium (Melford Ltd, Ipswich, UK). For germination of transgenic seed, agar media were supplemented with kanamycin (50 μg.ml^-1^), hygromycin B (29 μg.ml^-1^), or both antibiotics as appropriate. After transfer to soil, all plants were maintained in a growth room (Conviron Ltd., Winnipeg, Manitoba, Canada) under a 16 h photoperiod (200 μE.m^-2^.s^-1 ^of photosynthetically active radiation) at 22°C and 60% relative humidity.

### Generation of transgenic tobacco and *N. benthamiana *constitutively expressing wild-type and mutant *Aox1a *gene sequences

Adapting a method previously used [45], pDJSnAOX-E was generated by mutagenesis of the *Aox1 *cDNA insert of pDJSn [20] to replace the codon for the active site glutamate 221 with one for alanine using the oligonucleotides 5'-GAAGCTGAAAATGCCAGGATGCACCTCATGAC-3' and 5'-GTCATGAGGTGCATCCTGGCATTTTCAGCTTC-3', and the Stratagene Quikchange XL site-directed mutagenesis kit (http://www.stratagene.com/). *Agrobacterium tumefaciens *strain GV3101 cells were transformed with DNA for pDJSn or PDJSnAOX-E using the freeze-thaw method [62]. The leaf disc method [63] was used for *A. tumefaciens*-mediated transformation of *N. benthamiana *and transgenic *N. benthamiana *harboring the *35S:MtRDR1 *transgene [22]. Conditions used for transformation of *N. benthamiana *were similar to those used for tobacco [20], except that shooting and rooting media were modified to contain 1 μM 1-naphthaleneacetic acid. Transformed lines were screened for transgene incorporation and expression using PCR and RT-PCR, respectively. Immunoblot analysis was used to detect constitutive AOX production and altered alternative respiratory pathway capacity was determined using established methods [20,45,40,64]. Lines of doubly-transformed plants derived from *MtRDR1*-transgenic plants that had been super-transformed with pDJSn were also checked for expression of the MtRDR1 by RT-PCR and using *in vitro *RDR activity assays as described by Xie et al. [19].

### Virus strains, detection of infection and chemical treatments

TMV (Genus, *Tobamovirus*; Species, *Tobacco mosaic virus*) strain U1 [65] and PVX (Genus *Potexvirus*; Species *Potato virus X*) strain UK3 [66] were used in this study. Immunoblot detection of TMV and PVX using appropriate anti-coat protein sera followed a previously described method [67]. For quantification of PVX accumulation, leaf samples were homogenized as described by Murphy et al. [67] and used in double-antibody sandwich-ELISA [68] using reagents and antibodies and following instructions supplied by Bioreba (http://www.bioreba.ch/). Quantification was achieved with a standard curve using known amounts of purified virus. Data from transgenic plants were assessed for statistically-significant (*p *< 0.05) differences from non-transgenic controls using two-tailed t-tests [statistical software Genstat^® ^2010 Thirteenth Edition, ^© ^Lawes Agricultural Trust (Rothamsted Research), VSN International Ltd., Hemel Hempstead, UK.; [69]].

For whole-plant treatments with SA, five-to-six week old *N. benthamiana *plants were sprayed for four consecutive days with either a control solution [0.05% (w/v) ethanol] or 1 mM SA dissolved in 0.05% (w/v) ethanol before rub inoculation with PVX or TMV on one or two lower leaves as previously described for tobacco [11,20]. Chemical treatment of leaf tissue by infiltration was carried out as described by Gilliland et al. [20] using SA or antimycin A concentrations described in Results and figure legends.

## Authors' contributions

WSL and FSF carried out the experimental procedures, with additional experiments by JVL on PVX accumulation. JPC initiated and directed the research and wrote the manuscript. All authors read and approved the final manuscript.

## Supplementary Material

Additional file 1 **Detection of AOX-E protein expression and over-expression of AOX protein in transgenic *Nicotiana benthamiana *plant lines used in this study**. Immunoblot detection of AOX (A) or mutant AOX-E (B) present in non-transgenic (NT) *N. benthamiana *plants and T_2 _generation transformed plants belonging to various independent lines (numbered) harboring AOX or AOX-E transgenes expressed under the control of the 35S constitutive promoter. Equal amounts of Triton X-100 soluble proteins were denatured in the presence of 0.1 M dithiothreitol and subjected to immunoblot analysis using an anti-AOX monoclonal antibody. Anti-AOX binding was detected using anti-mouse immunoglobulin conjugated to horseradish peroxidase and a chemiluminescent substrate. A protein sample extracted from a plant of the Sn6 transgenic tobacco plant line, which over-expresses AOX (Murphy et al., 2004), served as the Positive Control. The major cross-reacting band in all cases corresponded in size (apparent Mr *c*.35kDa) to the reduced form of AOX. Pre-stained Mr markers were not visible on the X-ray film. All lines express AOX or AOX-E at much higher levels than the native AOX protein which is not detectable on this western blot. Click here for file

Additional file 2 **Image showing TMV-induced systemic symptoms of TMV on non-transgenic, control transgenic and MtRDR1-transgenic *N. benthamiana***. Non-transgenic (NT) *Nicotiana benthamiana *plants, plants transformed with an 'empty' transformation vector (Control) and transgenic lines constitutively expressing MtRDR1 (35S:MtRDR1: Reference 22) were inoculated with TMV U1 strain (0.05 ug/ml) and photographed four weeks later. Mock-inoculated NT plants are shown for comparison. The scale bar is 8 cm. Click here for file
