# Supplementary material for: Genetic modification of alternative respiration in Nicotiana benthamiana affects basal and salicylic acid-induced resistance to potato virus X
Source: BMC Plant Biol. 2011 Feb 28;11:41. doi: 10.1186/1471-2229-11-41 (PMC3058079; doi:10.1186/1471-2229-11-41)
Supplement: Additional file 1 — Detection of AOX-E protein expression and over-expression of AOX protein in transgenic Nicotiana benthamiana plant lines used in this study. Immunoblot detection of AOX (A) or mutant AOX-E (B) present in non-transgenic (NT) N. benthamiana plants and T2 generation transformed plants belonging to various independent lines (numbered) harboring AOX or AOX-E transgenes expressed under the control of the 35S constitutive promoter. Equal amounts of Triton X-100 soluble proteins were denatured in the presence of 0.1 M dithiothreitol and subjected to immunoblot analysis using an anti-AOX monoclonal antibody. Anti-AOX binding was detected using anti-mouse immunoglobulin conjugated to horseradish peroxidase and a chemiluminescent substrate. A protein sample extracted from a plant of the Sn6 transgenic tobacco plant line, which over-expresses AOX (Murphy et al., 2004), served as the Positive Control. The major cross-reacting band in all cases corresponded in size (apparent Mr c.35kDa) to the reduced form of AOX. Pre-stained Mr markers were not visible on the X-ray film. All lines express AOX or AOX-E at much higher levels than the native AOX protein which is not detectable on this western blot. [file 1471-2229-11-41-S1.PDF]

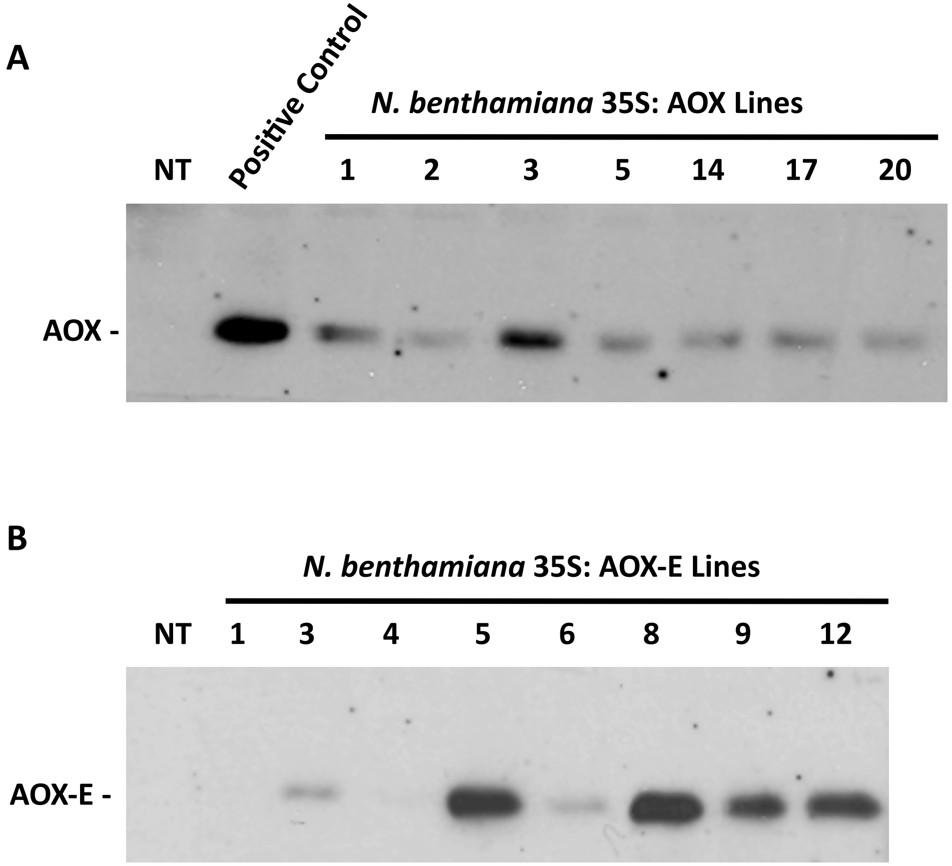

Additional File 1. Detection of AOX-E protein expression and over-expression of AOX protein in transgenic *Nicotiana benthamiana* plant lines used in this study. Immunoblot detection of AOX (A) or mutant AOX-E (B) present in non-transgenic (NT) *N. benthamiana* plants and T_2 generation transformed plants belonging to various independent lines (numbered) harboring AOX or AOX-E transgenes expressed under the control of the 35S constitutive promoter. Equal amounts of Triton X-100 soluble proteins were denatured in the presence of 0.1 M dithiothreitol and subjected to immunoblot analysis using an anti-AOX monoclonal antibody. Anti-AOX binding was detected using anti-mouse immunoglobulin conjugated to horseradish peroxidase and a chemiluminescent substrate. A protein sample extracted from a plant of the Sn6 transgenic tobacco plant line, which over-expresses AOX (Murphy et al., 2004), served as the Positive Control. The major cross-reacting band in all cases corresponded in size (apparent Mr *c*.35kDa) to the reduced form of AOX. Pre-stained Mr markers were not visible on the X-ray film. All lines express AOX or AOX-E at much higher levels than the native AOX protein which is not detectable on this western blot.
