# Supplementary material for: Genetic modification of alternative respiration in Nicotiana benthamiana affects basal and salicylic acid-induced resistance to potato virus X
Source: BMC Plant Biol. 2011 Feb 28;11:41. doi: 10.1186/1471-2229-11-41 (PMC3058079; doi:10.1186/1471-2229-11-41)
Supplement: Additional file 2 — Image showing TMV-induced systemic symptoms of TMV on non-transgenic, control transgenic and MtRDR1-transgenic N. benthamiana. Non-transgenic (NT) Nicotiana benthamiana plants, plants transformed with an 'empty' transformation vector (Control) and transgenic lines constitutively expressing MtRDR1 (35S:MtRDR1: Reference 22) were inoculated with TMV U1 strain (0.05 ug/ml) and photographed four weeks later. Mock-inoculated NT plants are shown for comparison. The scale bar is 8 cm. [file 1471-2229-11-41-S2.PDF]

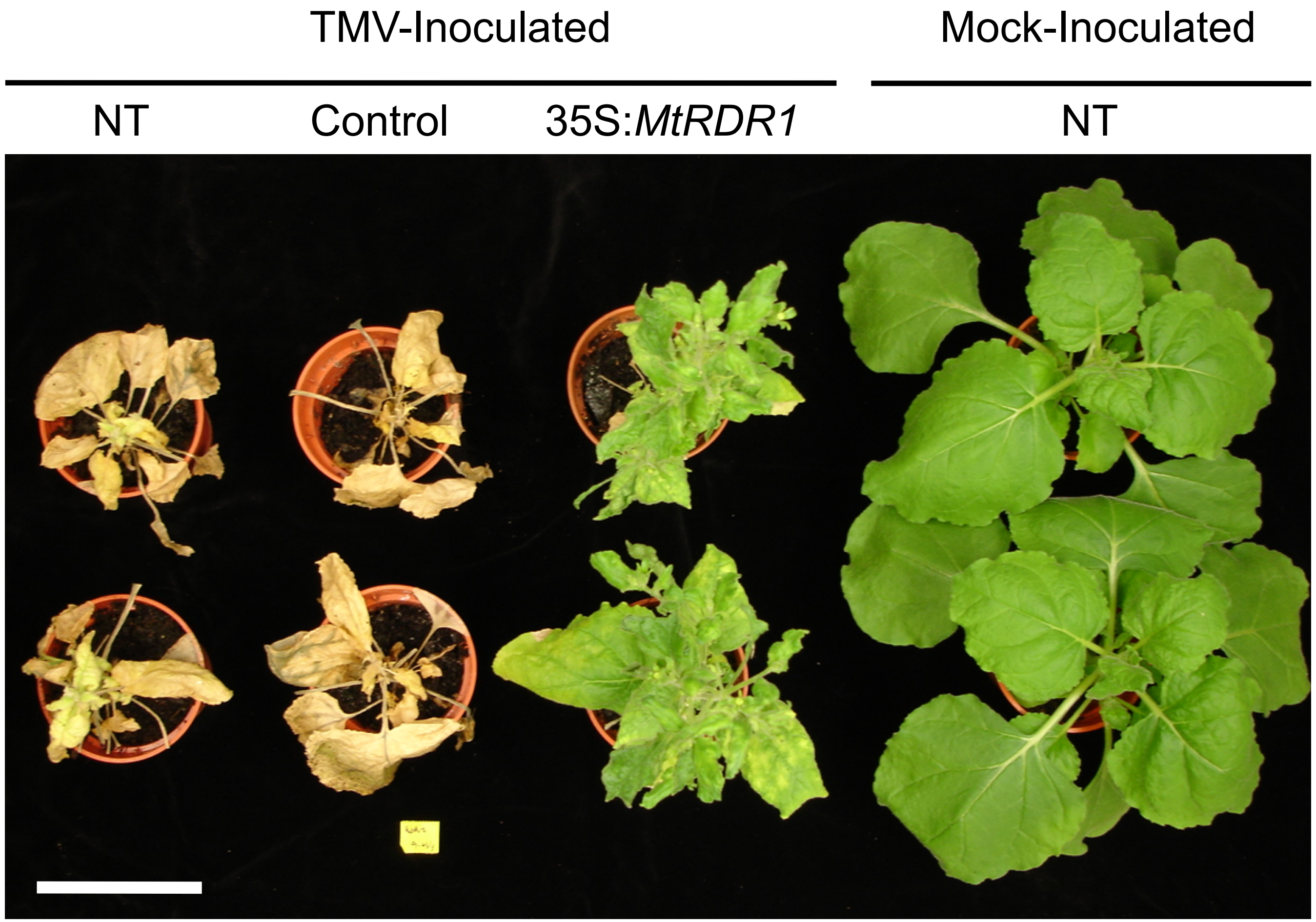

Additional file 2 – Image showing TMV-induced systemic symptoms of TMV on non-transgenic, control transgenic and MtRDR1-transgenic *N. benthamiana* Non-transgenic (NT) *Nicotiana benthamiana* plants, plants transformed with an 'empty' transformation vector (Control) and transgenic lines constitutively expressing MtRDR1 (35S:*MtRDR*1: Reference 22) were inoculated with with TMV U1 strain (0.05ug/ml) and photographed four weeks later. Mock-inoculated NT plants are shown for comparison. The scale bar is 8 cm.
